# Understanding catalyst behavior during *in situ* heating through simultaneous secondary and transmitted electron imaging

**DOI:** 10.1186/1556-276X-9-614

**Published:** 2014-11-14

**Authors:** Jane Y Howe, Lawrence F Allard, Wilbur C Bigelow, Hendrix Demers, Steven H Overbury

**Affiliations:** 1 Physical Sciences Directorate, Oak Ridge National Laboratory, 1 Bethel Valley Rd, TN 37831 Oak Ridge, USA; 2 Department of Materials Science and Engineering, University of Michigan, 1221 Beal Avenue, Ann Arbor, MI 48104, USA; 3 Department of Mining and Materials Engineering, McGill University, 845 Rue Sherbrooke Ouest, Montreal, QC H3A 2B2, Canada; 4 Hitachi High-Technologies Canada Inc, 89 Galaxy Blvd, Toronto, ON M9W 6A4, Canada

**Keywords:** Scanning transmission electron microscopy, Scanning electron microscopy, Catalyst, Phase transformation, *In situ* characterization

## Abstract

By coupling techniques of simultaneous secondary (SE) and transmitted electron (TE) imaging at high resolution in a modern scanning transmission electron microscope (STEM), with the ability to heat specimens using a highly stable MEMS-based heating platform, we obtained synergistic information to clarify the behavior of catalysts during *in situ* thermal treatments. Au/iron oxide catalyst 'leached' to remove surface Au was heated to temperatures as high as 700°C. The Fe_2_O_3_ support particle structure tended to reduce to Fe_3_O_4_ and formed surface terraces; the formation, coalescence, and mobility of 1- to 2-nm particles on the terraces were characterized in SE, STEM-ADF, and TEM-BF modes. If combined with simultaneous nanoprobe spectroscopy, this approach will open the door to a new way of studying the kinetics of nano-scaled phenomena.

## Background

Our ability to image surface and bulk features of nanomaterials plays an important role in the field of nano-scaled materials research. Even more desirable (especially for the study of catalytic materials) is the capability to simultaneously image morphological and structural changes that occur on the surface and within the bulk during *in situ* heating. Scanning electron microscopy (SEM) is by far the most widely used technique for imaging the surfaces of materials. Standard SEMs used for imaging bulk materials (i.e., samples installed below the final imaging lens) do not have a resolution high enough to clearly reveal the smallest nanoparticles such as catalysts. SEMs capable of in-lens operation have given sub-nanometer image resolution at a relatively low accelerating voltage, but the majority lack the capability for transmission electron microscopy. A modern scanning transmission electron microscope (STEM), operating at 60 to 300 kV (e.g., the Hitachi HF-3300 STEM/TEM (Hitachi, Tokyo, Japan)), can routinely provide imaging in the 0.2-nm range in both TEM and STEM imaging modes. Bulk crystal lattice structure is imaged with a 0.1-nm information limit in bright-field (BF) TEM mode and about 0.2-nm resolution in BF and annular dark-field (ADF) STEM modes. SE imaging resolution is limited by specimen-beam interaction effects, but resolution at the 1-nm level is achieved, even at elevated temperatures as shown in the present study. We report herein a simultaneous SE and TE study of morphological evolution of an Au/iron oxide catalyst during *in situ* heating in an Hitachi HF-3300 STEM/TEM operated at 300 kV. This microscope permits rapid and facile switching between TEM and STEM operating modes. In addition to the standard ADF and BF detectors that are fitted on most STEMs, this instrument also comes equipped with a secondary electron (SE) detector. Because it has a cold-field emission electron gun with an energy spread of <0.4 eV, the small probe size (approximately 0.2 nm) and the high-efficiency SE detector lead to an SE imaging resolution at better than 0.5 nm. It was of special interest in the present study to characterize the ability to image in SE mode at high temperatures, where light emitted from the heating device would typically (in conventional SE mode using a standard heater in a SEM [1,2]) cause the SE detector to be adversely affected and make high resolution imaging impossible. It was found that the particular characteristics of the Protochips Aduro™ (Protochips Inc., Raleigh, NC, USA) heating technology used in this study allows high resolution imaging in STEM-SE mode, even at temperatures as high as 700°C.

Environmental TEM and STEM have been used for catalysis research for decades [3-10]. Notably, Gai and Boyes pioneered the development of environmental cell and heating stage for *in situ* study of catalysts [3-5]. They achieved sub-Ångström resolution using the aforementioned setup in a TEM/STEM with double correctors [8-10]. Our work was carried out using a TEM/STEM without an aberration corrector. The goal of this work was to demonstrate the advantage of obtaining surface and bulk information of catalysts by simultaneous imaging using both transmitted (ADF, TE) and secondary electron signals (SE).

In this study, we have selected gold nanoparticles on hematite support as the material of interest, because gold nanoparticles have generated considerable interest for catalytic applications for certain oxidation reactions and selective hydrogenation reactions [11-14]. Recently, a series of Au catalysts supported on Fe_2_O_3_ (hematite) were characterized with atomic resolution during elevated temperature treatments, using Protochips Aduro *in situ* heating technology in an aberration-corrected STEM (JEOL 2200FS, JEOL, Tokyo, Japan) fitted with a CEOS hexapole corrector (CEOS GmbH, Heidelberg, Germany) on the probe-forming lenses) [15-17]. In order to extend the understanding of the behavior of Au species during heating, we carried out this simultaneous SE and TE imaging study in a sample of the Au/Fe_2_O_3_ system selected from the prior study [17,18].

## Methods

The microscopy was carried out using the Hitachi HF-3300 TEM/STEM at Oak Ridge National Laboratory. This instrument is fitted with a SE detector (using a photomultiplier tube), in addition to both BF and high-angle annular dark-field (HAADF) detectors for STEM imaging, and a Gatan 2 k × 2 k Ultrascan CCD camera (Gatan Inc., Pleasanton, CA, USA) for conventional TEM imaging. The advantage of TEM recording during *in situ* heating is that it provides shorter exposure times (approximately 1 s) relative to the slower scans required by SE in the STEM modes. This allows higher accuracy and more reliable analysis of the atomic structure via computed diffractograms (sample drift and scan artifacts can strongly affect STEM diffractograms). The column vacuum was maintained at 4.8 × 10^−6^ Pa even during *in situ* heating.

The capability of SE imaging in the Hitachi HF-3300 at the high temperatures afforded by the Protochips Aduro heater system is an added benefit for complementary analysis of catalyst structure and behavior during elevated temperature treatments. The MEMS-fabricated Aduro heater devices have a nominal 500 × 500 μm^2^ thin ceramic membrane on the order of 100-nm thick supported over a window in a silicon chip. The ceramic membrane has a pattern of 6-micron diameter holes over which is suspended a holey carbon support film (e.g., C-flat, Protochips, Inc.). In this study, all heating experiments were conducted under high-vacuum conditions in the microscope column. The heater device fits onto a special-made specimen holder, with electrical leads to provide power from a Keithley 2611A source meter (Keithley Instruments, Inc., Cleveland, OH, USA). The profile of temperature as a function of input current is calibrated by the manufacturer, using an optical pyrometer while heating the device in a vacuum chamber, thus allowing estimation of temperature of the sample deposited on it, up to 1,200°C. Owing to its miniature heated volume, the heating chip has minimal thermal drift and a near instantaneous temperature response at a heating/cooling rate on the order of 10^6^°C/s [15].

An Au/Fe_2_O_3_ (hematite) catalyst with nominal 2 wt% Au loading, acquired from World Gold Council (WGC Ref. No. 60C), was leached in sodium cyanide solutions at pH 12, to remove the weakly bound surface Au species, and was shown to contain 0.7 wt% Au after the leaching process [15]. The TEM specimen was prepared by depositing dry powder onto an Aduro device and then simply shaking off the excess. Imaging experiments were conducted first at room temperature, and the sample was then heated for 10 min at 250°C for stabilization (prior work had indicated that heating at 250°C for up to 30 min did not measurably affect the morphology of Au species in the support) [16,17]. The sample was imaged during further heating at target temperatures of 500°C, 600°C, and 700°C. It was estimated that it reached the targeted temperature in no more than 10 s. This estimation is derived from a separate study which used powdery materials with known melting point.

## Results and discussions

### Conventional TEM imaging (TEM-BF)

**Figure 1:**
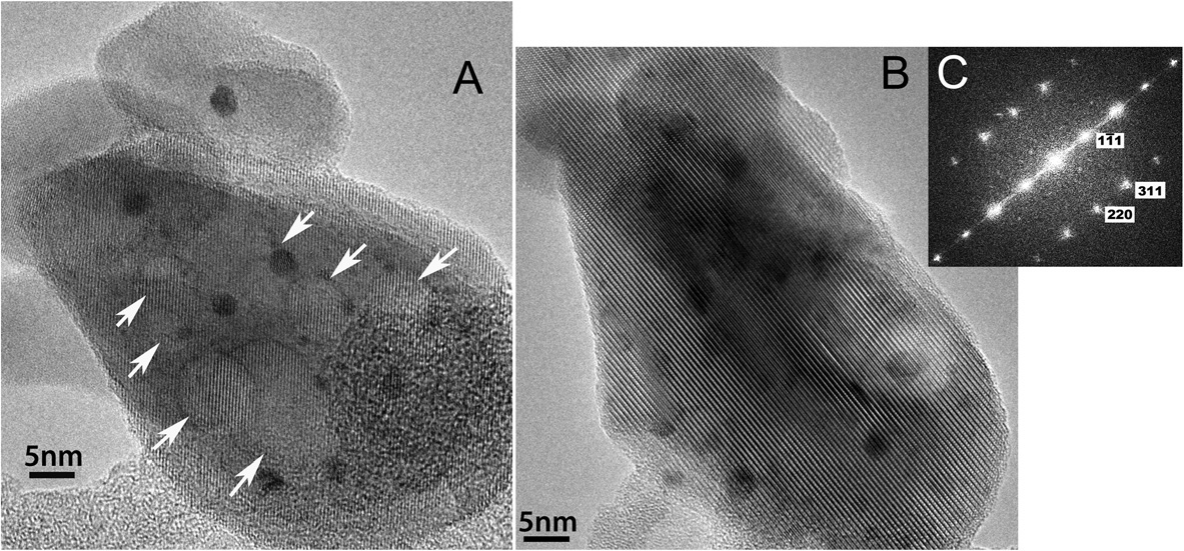
**Bight-field transmission electron micrographs recorded in TEM mode. (A)** Of leached Au/Fe_2_O_3_ (hematite) catalyst at room temperature, showing voids and Au nanoparticles; **(B)** same support particle imaged at 500°C, revealing Au particle growth and void shrinkage; **(C)** diffractogram from heated particle, consistent with a <112 > zone axis of magnetite (Fe_3_O_4_). The dim, fine spots in diffractogram were from that of hematite.

The principal features observed in STEM-HAADF and STEM-BF images in the earlier studies of the leached catalyst [17] were voids in the hematite support particles, as well as Au nanoparticles and highly dispersed Au species internal to the support and coating the void surfaces. This as-leached catalyst is shown in the BF image of Figure 1A, with a number of voids indicated by arrows, and several nanoparticles in the 2- to 3-nm size range are evident. Many Au nanoparticles in the 1 nm and smaller range are also evident in the BF image, consistent with the HAADF images shown by Allard et al. [15-17] In their study, at 250°C, no significant changes were observed for the voids (or Au species) over time. But at 500°C, the voids diminished in size and gradually disappeared in a time period of 10 min, while internal Au species diffused to the surfaces to form new 1- to 2-nm Au nanoparticles. The TEM-BF image of the same area obtained after several minutes of heating at 500°C is shown in Figure 1B. The voids were observed to shrink somewhat during this period of heating at 500°C, an observation consistent with earlier results. Some void structure remained after this short heating time, but no surface Au nanoparticles were observed until further heating was conducted. The support particle morphology apparently changed due to the void shrinkage effects but primarily due to a significant rotation of the particle during the thermal treatment. This rotation brought the iron oxide particle into a strong zone-axis alignment, as shown by the lattice structure visible in the image and by the diffractogram from this image of the particle that shows periodicities consistent with *d*-spacings for the <112 > zone axis orientation of magnetite (Fe_3_O_4_). This indicates that the hematite particle was reduced during heating to 500°C in vacuum.

In the past, reduction of hematite to magnetite at elevated temperatures in vacuum was studied using a thermomagnetic analysis method. Shive and Diehl suggested that partial reduction to magnetite occurs on the surfaces of micron-sized hematite crystallites in a vacuum of 1.4 × 10^−3^ Pa starting at 350°C [19]. Absalyamov and Mulyukov measured the value of saturation magnetization of submicron-grained hematite in a 1.4 × 10^−3^ Pa vacuum during heating and cooling up to 750°C [20]. They attributed the sharp increase of saturation magnetization near 500°C to the reduction of hematite to magnetite and further suggested that nano-crystallites of hematite could not exist in vacuum at elevated temperatures. Neither group provided any direct evidence of such a phase transition, citing that the fraction of formed magnetite is too low to be detected by X-ray diffraction. Our *in situ* TEM study, for the first time, presents the direct evidence that the nano-sized hematite completely converts to magnetite at 500°C in a vacuum of 4.8 × 10^−6^ Pa. Our experimental condition was at higher vacuum than the previous two groups. The higher vacuum level may contribute to a more favorable condition for the reduction of hematite nanocrystals. Because this is an Au/Fe_2_O_3_ (hematite) material system, it is also worth exploring whether the presence of Au nanoparticles and finely dispersed species in the hematite lattice might play a role in the reduction of the hematite. In further work, similar heating experiments will be carried out using equivalent hematite powders without the presence of Au.

**Figure 2:**
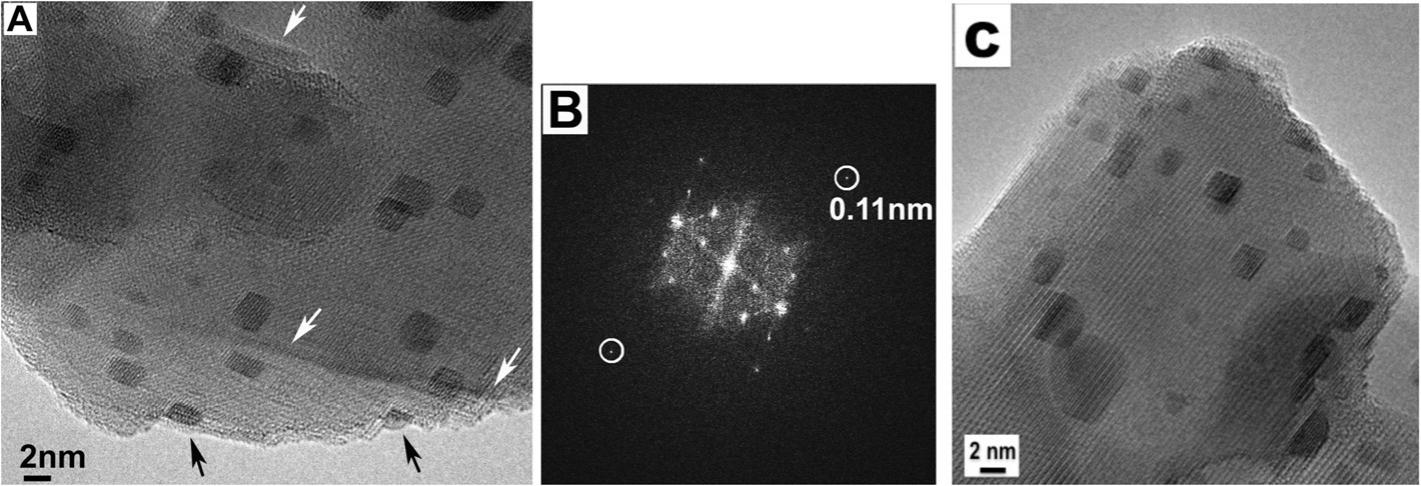
**TEM-BF image, diffractogram, and movie clip. (A)** TEM-BF image recorded at 600°C, showing the emerging Au on the surfaces (by black arrows) and the developing steps on Fe_3_O_4_ (by white arrows); **(B)** diffractogram showing information transfer to 0.11 nm; and **(C)** movie clip constructed from the 1-s exposure still images.

Raising the heating temperature to 600°C caused the appearance of steps on the magnetite support, as marked by the white arrows in Figure 2A. The Au particles grew larger to the 3- to 5-nm range in less than 3 min. Internal voids were fully shrunk and disappeared, and the Au nanoparticles appeared on the surface steps, as are marked by the black arrows. The information transfer from this image was calculated from the diffractogram of Figure 2B to be about 0.11 nm [21]. In Figure 2C, a movie clip is constructed from the TEM-BF images taken at 600°C that revealed the epitaxial growth of gold on iron oxide (Additional file 1).

### STEM imaging results

**Figure 3:**
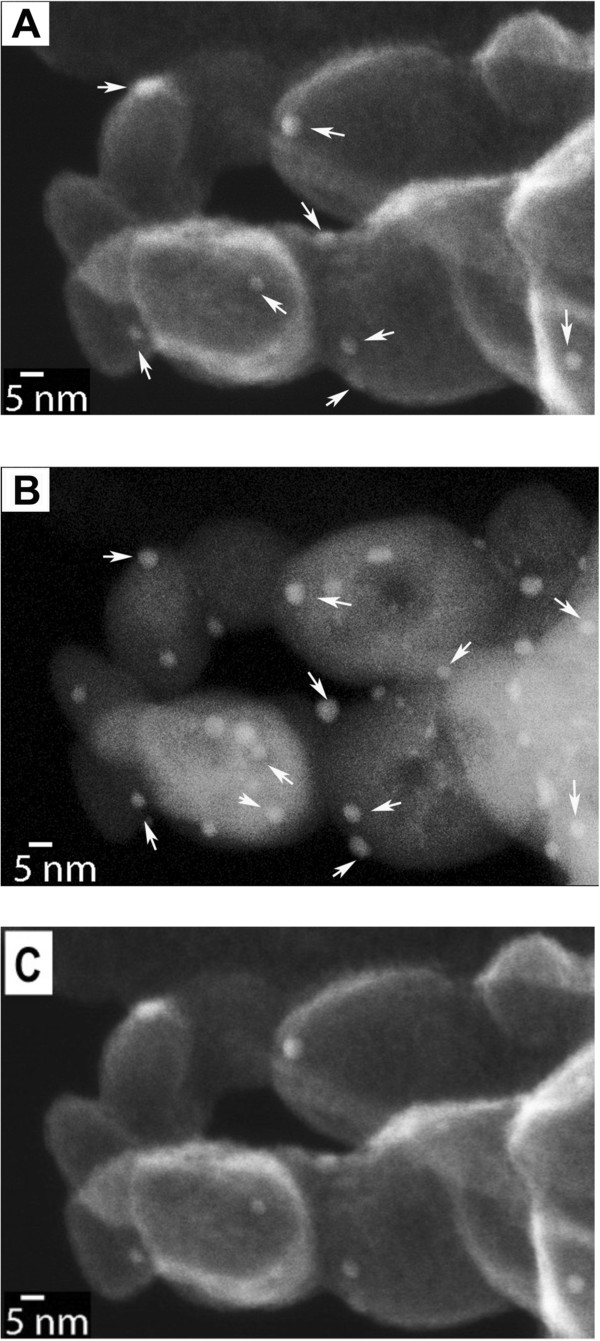
**Simultaneous acquisition of a pair of *unprocessed *STEM-SE (A) and ADF images (B) video clip (C).** Taken at 500°C; movie clip can be viewed online only.

In Allard et al.’s earlier study of this materials system, whether the Au particles were on the surface or interior of the iron oxide support were deduced from the through-focus HAADF image series. The high resolution in Z (depth sectioning) in their study was made possible by the large probe incidence semiangle in the aberration-corrected instrument [17]. It is a time-consuming approach which required careful interpretation of a series of ADF images taken only from an aberration-corrected instrument. Unlike TE imaging, SE imaging has the advantage of being surface-sensitive and can thereby better reveal topographic information. Figure 3 presents a pair of *unprocessed*, simultaneously acquired STEM-SE and STEM-ADF images taken at 500°C and 1.2 million times direct magnification. The SE image in Figure 3A reveals the Au particles as lighter-contrast dots (marked by white arrows), providing a better three-dimensional impression of the area than that of the ADF micrograph in Figure 3B. There are many more bright spots (Au) in Figure 3B, suggesting the extra spots are either within the iron oxide or on the bottom surfaces of the support. The internal voids were revealed only in the ADF micrograph. Comparing the pair of images, the information from both surface and bulk are readily obtained. Movies of 30 frames/s were also recorded through the NTSC port provided on the scanning control system. For instance, a movie clip in Figure 3C captures the structural change during heating; the instant switching among SE, ADF, and BF signals provides a powerful means for studying dynamic processes that occurred on the surface and bulk of the catalyst (Additional file 2).

The spatial resolutions of Figure 3A,B were determined with the SMART-J method [22,23]. This method uses the Fourier transform (FT) of the image to separate the contribution of the signal (object) and the noise in the FT image. A resolution of 1.5 nm was determined for the SE image (Figure 3A), and a resolution of 1.6 nm was determined for the ADF image (Figure 3B). The particle sizes were measured from intensity line scans extracted from the image. The size was defined by the full-width-half-maximum (FWHM) of the intensity peak corresponding to the particle. An average size of 3.3 ± 0.4 nm was obtained after the analysis of five particles. No difference in size was observed between the SE and ADF images.

**Figure 4:**
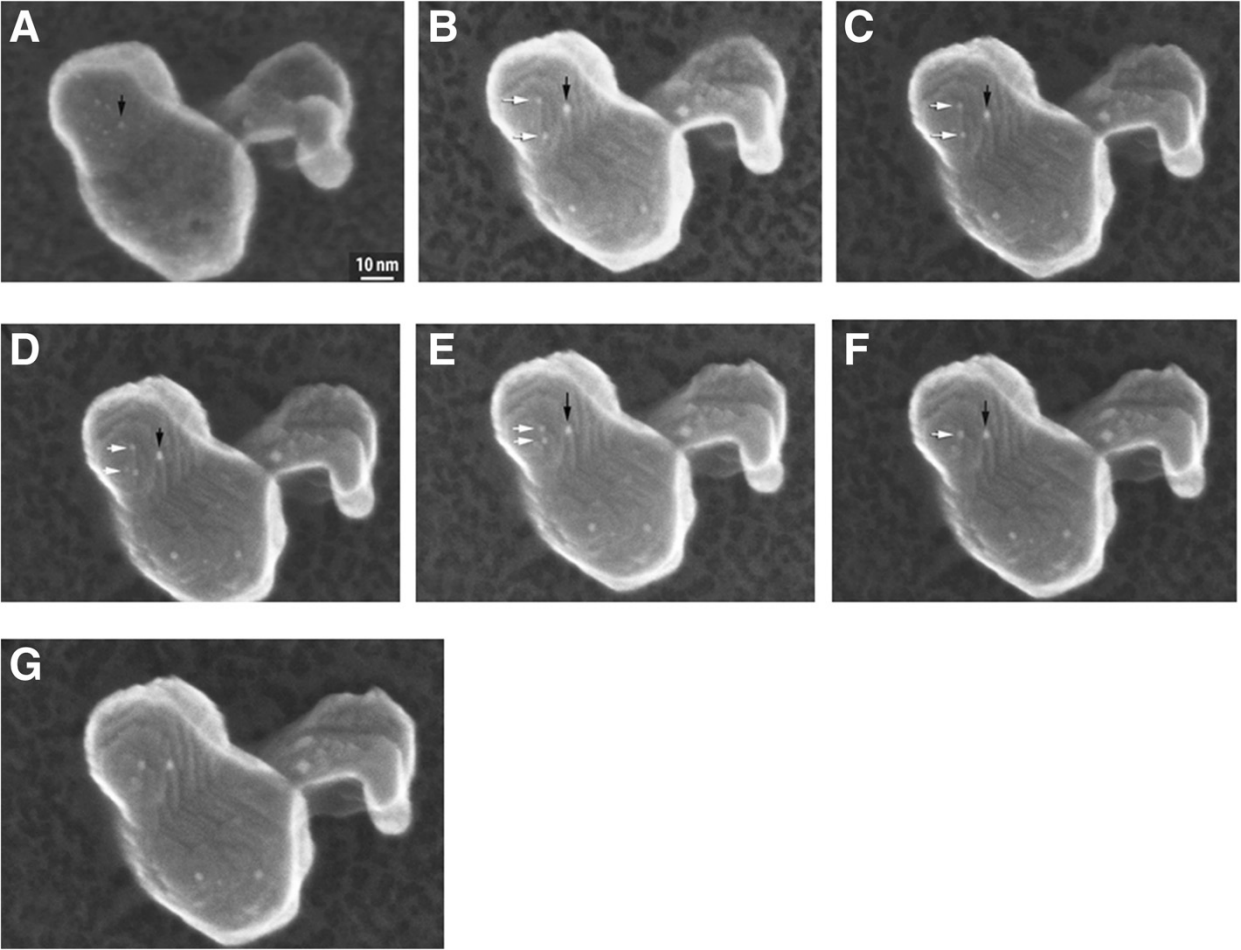
**Sequence of SE images recorded at 300 kV in the Hitachi HF-3000.** With the same support particle held at 700°C, several Au nanoparticles are visible on the surface; **(A)** initial image at the start of sequence taken at room temperature; **(B-F)** SE images from 30 to 200 s at 700°C show development of facets on the support surface and movement and coalescence of nanoparticles, as arrowed; and **(G)** a movie clip constructed from the images **(A)** through **(F)**.

Further heating at 700°C resulted in additional morphological changes. Figure 4 shows a series of *unprocessed* SE images taken at 700°C. Figure 4A shows a relatively smooth surface at the beginning of the 700°C heating cycle and the presence of a number of 1 nm Au particles. Figure 4B,C,D,E,F, acquired over 3 min, shows that the support surface developed terraces and facets and that some Au particles migrated on the terraces to coalesce into larger particles (e.g., as arrowed). A movie included in Figure 4G was constructed from the still images (Additional file 3). A spatial resolution of 1.1 nm was determined with SMART-J method for all images in Figure 4. Heating of the specimen did not affect the resolution of the microscope, and although heating to 700°C caused the membrane to glow in the visible light range, no significant effect on the quality of the SE image was observed. This can be attributed to the tiny mass of the Aduro heating element, which does not emit enough photons to affect the electron detector, as opposed to the effect of light on secondary electron imaging when more standard heating stages employing bulk furnaces are used [1,2]. The size of the particle indicated by a black arrow was also measured on all images. An average size of 2.3 ± 0.3 nm was obtained. The size was not affected by heating, indicating it was isolated on the terrace of the iron oxide.

Secondary electron micrographs are formed using secondary electrons (SE) and backscattered electrons (BSE). In a recent study by Zhu et al. [24], conducted using an essentially identical secondary detector, the ratio of SE and BSE was found to be in the range of 85% to 90% (SE) to 15% to 10% (BSE) at 200 kV. We estimated the fraction of secondary electrons exceeds 95% from a test using an atomically smooth silicon nitride film of 50-nm thick: the film facing the electron incident beam was clean. On the opposite side, we deposited Li_3_FePO_4_. There was absolutely no detectable contrast from the SE image. This test demonstrated that the signals from Figure 3A and Figure 4 are mostly from secondary electrons emitted from less than 50 nm depth. We further calculated that at 700°C the SE image resolution was 1.1 nm.

## Conclusions

In summary, we have demonstrated that 1.1-nm spatial resolution in secondary electron imaging can be achieved even during *in situ* heating up to 700°C using a conventional TEM/STEM. We also have shown that information transfer in TEM imaging is about 0.14 nm at 600°C. Such a combined SEM and TEM *in situ* study is useful for nanomaterials research because information from the surface via SEM imaging and bulk via TEM/STEM imaging can be simultaneously obtained. If combined with simultaneous nanoprobe spectroscopy (e.g., energy dispersive X-ray spectroscopy and energy-loss electron spectroscopy), this approach will open the door to a wide range of applications, such as studying the kinetics of nano-scaled phenomena.

## Competing interests

The authors declare that they have no competing interests.

## Authors’ contributions

JYH, LFA, and SHO came up with the research idea and wrote up the manuscript. JYH and LFA carried out experiments. WCB was responsible for the instrumentation. HD performed the SE imaging analysis and data interpretation. All authors read and approved the final manuscript.

## Supplementary Material

Additional file 1 **Movie clip of Figure 2c.** A movie clip constructed from the 1-s exposure still TEM-BF image recorded at 600°C. Click here for file

Additional file 2 **Movie clip of Figure 3c.** Simultaneous acquisition of a pair of *unprocessed* STEM-SE and ADF imaging at 500°C. Click here for file

Additional file 3 **Movie clip of Figure 4g.** A movie clip constructed from a series of SE images recorded at 300 kV at 700°C. Click here for file
